# Orbit Determination for Continuously Maneuvering Starlink Satellites Based on an Unscented Batch Filtering Method

**DOI:** 10.3390/s25134079

**Published:** 2025-06-30

**Authors:** Anqi Lang, Yu Jiang

**Affiliations:** 1 State Key Laboratory for Strength and Vibration of Mechanical Structures, School of Aerospace Engineering, Xi’an Jiaotong University, Xi’an 710049, China; 2 State Key Laboratory for Space-System Operation and Control, Xi’an Satellite Control Center, Xi’an 710049, China; jiangyu.2012@tsinghua.org.cn

**Keywords:** orbit determination, low-thrust maneuvers, Starlink satellites, orbital dynamics model, unscented transformation, batch filtering

## Abstract

Orbit determination for non-cooperative low Earth orbit (LEO) objects undergoing continuous low-thrust maneuvers remains a significant challenge, particularly for large satellite constellations like Starlink. This paper presents a method that integrates the unscented transformation into a batch filtering framework with an optimized rho-minimum sigma points sampling strategy. The proposed approach uses a reduced dynamics model that considers Earth’s non-spherical gravity and models the combined effects of low-thrust and atmospheric drag as an equivalent along-track acceleration. Numerical simulations under different measurement noise levels, initial state uncertainties, and across multiple satellites confirm the method’s reliable convergence and favorable accuracy, even in the absence of prior knowledge of the along-track acceleration. The method consistently converges within 10 iterations and achieves 24 h position predictions with root mean square errors of less than 3 km under realistic noise conditions. Additional validation using a higher-fidelity model that explicitly accounts for atmospheric drag demonstrates improved accuracy and robustness. The proposed method can provide accurate orbit knowledge for space situational awareness associated with continuously maneuvering Starlink satellites.

## 1. Introduction

In recent years, the rapid deployment of low Earth orbit (LEO) satellite mega-constellations, typically represented by Starlink, has led to a marked increase in the number of low-orbit space targets. As of 28 April 2025, the number of operational Starlink satellites in orbit was reported to be 7239 according to Jonathan’s Space Pages at https://planet4589.org/space/con/star/stats.html (accessed on 28 April 2025). In addition to their large population, Starlink satellites are characterized by frequent orbital maneuvers. Most Starlink satellites are initially injected into orbits at an approximate altitude of 300 km. Subsequently, the satellites ascend to operational altitudes above 500 km using continuous low-thrust electric propulsion. This process often involves several temporary parking phases. Both the orbit-raising and orbit-lowering phases generally last several months, while the on-station phase can extend for several years. Compared to infrequently maneuvering space targets, Starlink satellites experience substantially larger orbit determination (OD) and prediction errors. According to the statistical results presented in [1], for uncontrolled LEO satellites, the 7-day orbit prediction error using TLE data with SGP4 is approximately 10 km, mainly in the along-track direction. For Starlink satellites during the orbit-raising phase, the 1-day prediction error for the V1 version satellites can reach several tens of kilometers [2]. For the V2mini version satellites, which are equipped with higher thrust, the 1-day prediction error can be even larger, reaching several tens or even over a hundred kilometers. This is primarily due to the difficulty in accurately characterizing their thrust force models and maneuvering schedules. This consequently gives rise to a set of challenges concerning space situational awareness, such as tracking and cataloging space targets and collision warning.

In the domain of satellite orbit determination, estimation algorithms play an important role. Among the most widely employed are batch processing methods, which include the least squares method (LSM) [3,4,5,6,7], and sequential processing methods, represented by the Kalman filtering (KF) and its variants [8,9,10,11]. The Batch Least Squares (BLS) method is a classical OD approach. This method linearizes the nonlinear dynamical system and measurement equations through a Taylor series expansion, neglecting higher-order terms. The OD procedure involves computing partial derivatives of both the dynamics system and measurement equations with respect to the state variables. By simultaneously processing a complete set (or a substantial portion) of observations collected over a defined time span, BLS can estimate the orbital parameters with high accuracy under good initial guesses. However, as system nonlinearity and the number of state variables increase, the calculation of the required partial derivatives becomes more complex. In situations with strong nonlinearity and sparse or limited observations, the BLS method may encounter convergence issues, potentially resulting in OD failure. Among the various Kalman filter variants, the Unscented Kalman Filter (UKF) distinguishes itself from traditional linearization-based approaches by applying the unscented transformation (UT) within the standard Kalman filtering framework. Employing a set of sigma points, the UKF approximates the posterior state distribution, allowing it to address nonlinear system and measurement models directly without computing partial derivatives. This approach can capture higher-order statistics and enhance filtering performance under nonlinear conditions. However, the performance of the UKF is sensitive to the choice of sigma points, weighting parameters, and initial assumptions, including prior variance and process noise [11,12]. Like other sequential estimation methods, the UKF updates the system state vector in real time through a prediction–correction cycle, where the prediction step relies on orbital dynamics and the correction step incorporates newly acquired measurements to refine the estimate. However, in the context of LEO space targets undergoing continuous low-thrust maneuvers, the interval between successive observation arcs may become prolonged—often exceeding ten hours. In such cases, the unknown low-thrust acceleration introduces significant model uncertainty, leading to diffusion of the state covariance matrix during propagation. This not only slows convergence but may also result in filter divergence [13].

For orbit determination of maneuvering space targets, a variety of methods have been developed based on variants of LSM and KF [14,15,16,17,18]. These approaches can be broadly classified into 3 categories: reinitialization of orbit determination, maneuver reconstruction, and filtering-based approach [17,19,20]. The reinitialization approach [21] uses only post-maneuver measurements and does not explicitly model the maneuver itself. While simple, it fails to account for maneuver dynamics. The maneuver reconstruction approach [17,22,23] seeks to estimate detailed maneuver parameters, such as thrust magnitude, direction, and timing (i.e., maneuver start and end times). Although this method can provide more physically accurate representations of the maneuvering behavior, the high computational cost limits its application to scenarios involving frequent maneuvers or multiple targets. Moreover, it is unsuitable for orbit prediction (OP) when future maneuver plans are unknown or unavailable. In contrast, the filtering-based approach, which is grounded in KF and its nonlinear variants, offers a more flexible framework for estimating and predicting the orbits of non-cooperative targets. This class of methods avoids the need for detailed maneuver profiles by incorporating an equivalent or compensated dynamics model.

The dynamic system with unknown maneuvers can be modeled in different ways. To model the maneuvers of geostationary (GEO) satellites, Xu et al. [15] employed Newton’s high-resolution differential formulation in combination with polynomial fitting. In their approach, the maneuver acceleration is treated as a residual term, obtained by subtracting the modeled acceleration from the interpolated total acceleration derived from the estimated satellite states. Zhou et al. [20] adopted a polynomial representation model for unknown maneuvers and introduced a transformation technique that reduces sensitivity to higher-order temporal terms. This approach enhances the convergence and robustness of the OD process. Based on the observed orbital characteristics of climbing Starlink satellites, Li et al. [2] adopted a model that assumes a constant but unknown acceleration over multiple days. They developed a recursive OD and OP framework to enable tracklet-to-object association. In the context of tracking maneuvering targets, Singer et al. [24] and Whang et al. [25] modeled the target maneuvers as a first-order Markov process. Ko and Scheeres [19,26] proposed the Thrust-Fourier-Coefficient (TFC) model to represent the averaged variations in classical Keplerian elements caused by maneuvers. Zhang et al. [27] further improved this model by replacing Keplerian elements with nonsingular orbital elements.

Besides the maneuver modeling, the performance of the filtering-based OD methods largely depends on the estimation of the initial state and the characterization of noise statistics. In general, initial position and velocity are relatively straightforward to obtain. These can be derived from existing ephemeris data of cataloged satellites or computed through initial orbit determination (IOD) techniques based on early-stage observational data. In contrast, initializing unknown maneuver accelerations is significantly more challenging, especially in the absence of prior information. To address the challenge of initializing maneuver accelerations, Li et al. [2] proposed an empirical formula based on the TLE B* parameter. This approach, validated on a large set of climbing Starlink satellites, was shown to significantly improve filter convergence. Nevertheless, our experiments reveal that this method no longer performs effectively for the newer generation of Starlink satellites, such as the V2 Mini version, indicating limited generalizability across evolving spacecraft platforms. With regard to noise modeling, accurate specification of process and measurement noise statistics is important to ensuring filter stability and estimation accuracy. Zhou et al. [11] extended the concept of uncertainty propagation to the prediction of measurement uncertainty, thereby improving data association performance. However, the maneuvering case remains unexplored in their research. Guo et al. [12] investigated the impact of noise variance on Kalman filter performance and proposed using genetic algorithms (GA) to optimize both process and measurement noise variances. Their results showed that this optimization reduces estimation errors by avoiding arbitrary parameter selection. Zhang et al. [27] incorporated the process noise covariance associated with the TFC model offset into an iterative extended Kalman filter (EKF), in order to compensate for the unmodeled dynamics and improve the OD accuracy. The limitation of this method is that the process noise uncertainty must be estimated offline in advance. For more general maneuvering scenarios, Jia-Richards et al. [28] developed a thrust estimation method based on ensemble Kalman updating. This approach avoids linearizing the spacecraft dynamics and relaxes Gaussian assumptions on parameter and measurement noise distributions. Despite its advantages, the method relies on prior knowledge of thrust activation and termination times, which are often unavailable for non-cooperative targets. Considering the unknown or time-varying measurement noise characteristics, Rong and Wang [29] proposed an adaptive noise factor approach based on the UKF, which dynamically adjusts the measurement noise covariance matrix. Although this approach allows the noise factor to adapt to changing conditions, an upper bound must still be manually defined for each specific case.

To address the challenges of orbit determination for Starlink satellites—non-cooperative targets with unknown maneuvering parameters—this study proposes a dedicated framework. The focus is on the orbit-raising phase, where satellites perform frequent low-thrust maneuvers and observational data is sparse. The proposed framework employs a batch processing method combined with UT to estimate the satellite’s state vectors (position and velocity) as well as the equivalent thrust acceleration at selected epochs. By making full use of available observations, the method improves the stability and accuracy of orbit determination. It avoids dependence on predefined process noise models by achieving convergence through iterative refinement. Moreover, the implementation of UT via deterministic sampling effectively addresses measurement-state nonlinearities. Unlike the conventional symmetric sampling strategy used in [30], this study adopts the rho-minimum sigma set strategy [31], which generates a reduced number of sigma points. This modification can significantly reduce computational cost while maintaining estimation performance in high-dimensional augmented state spaces where position, velocity, thrust acceleration, and perturbation-related parameters are estimated simultaneously.

The following part of this paper begins by introducing a reduced dynamics model based on the orbital characteristics of Starlink satellites during the orbit-raising phase. Building on this, an unscented batch filtering method is developed for orbit determination, incorporating the rho-minimum sigma set sampling strategy to improve computational efficiency. The proposed approach is systematically evaluated through numerical simulations under various measurement noise levels, initial state covariances, and across multiple climbing Starlink satellites. Its effectiveness is further assessed using a higher-fidelity dynamics model in which the atmospheric drag is explicitly accounted for.

## 2. Materials and Methods

### 2.1. Orbital Dynamics Model

#### 2.1.1. General Model

Taking the maneuver into account, the satellite’s equations of motion in the Earth-centered inertial (ECI) coordinate system can be generally formulated as follows:

(1) $$\left\{\begin{array}{l} \frac{dr}{dt}=v\\ \frac{dv}{dt}=-\frac{\mu }{r^{3}}r+a_{\mathrm{NS}}+a_{3B}+a_{\mathrm{AD}}+a_{\mathrm{SRP}}+a_{\mathrm{TD}}+a_{\mathrm{RE}}+a_{\mathrm{TH}} \end{array}\right.,$$

where ***r***, ***v*** are the position and velocity vectors of the satellite, *r* is the magnitude of ***r***, and *μ* is the Earth’s gravitational constant. $-\frac{\mu }{r^{3}}r$ denotes the central gravitational acceleration from the Earth, and indicates the thrust acceleration. The other terms of acceleration represent the perturbation ones, specifically, $a_{\mathrm{NS}}$—perturbation due to the Earth’s non-sphericity, $a_{3B}$—the gravitational attraction of the third body, such as the Sun and the Moon, $a_{\mathrm{AD}}$—atmospheric drag, $a_{\mathrm{SRP}}$—solar radiation pressure, $a_{\mathrm{TD}}$—tidal force, and $a_{\mathrm{RE}}$—relativistic effects. Given that maneuvers are usually executed in the local orbit frame, the thrust acceleration can be expressed by

(2) $$a_{\mathrm{TH}}=M_{RTN\to ECI}\left[\begin{array}{l} a_{R}\\ a_{T}\\ a_{N} \end{array}\right],$$

where $a_{R}$, $a_{T}$, $a_{N}$ are the radial, along-track, and cross-track components of the thrust acceleration in the radial-transverse-normal (RTN) frame, $a_{RTN}=\left[a_{R}\;a_{T}\;a_{N}\right]$. $M_{RTN\to ECI}$ is the coordinate transformation matrix that maps vectors from the RTN frame to the ECI frame:

(3) $$M_{RTN\to ECI}=\left[\frac{r}{\left|r\right|},\;\frac{\left(r\times v\right)\times r}{\left|\left(r\times v\right)\times r\right|},\;\frac{r\times v}{\left|\left(r\times v\right)\right|}\right],$$

where ***r***, ***v*** are column vectors with dimensions 3 × 1.

#### 2.1.2. Reduced Dynamics Model for Climbing Starlink Satellites

**Figure 1:**
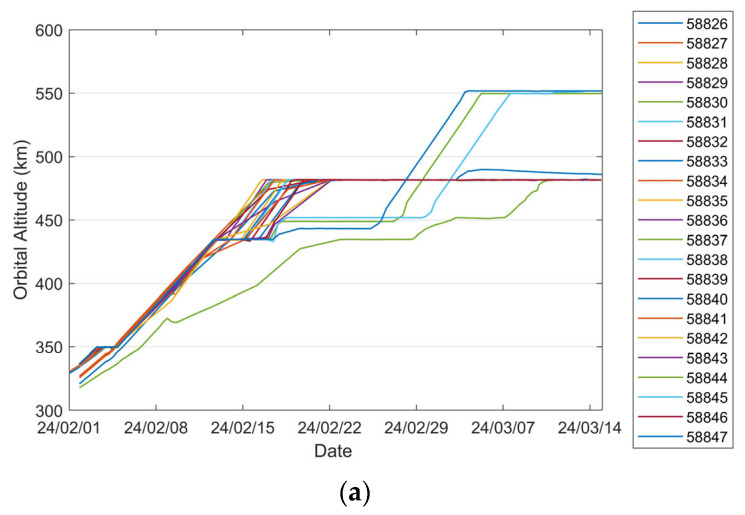

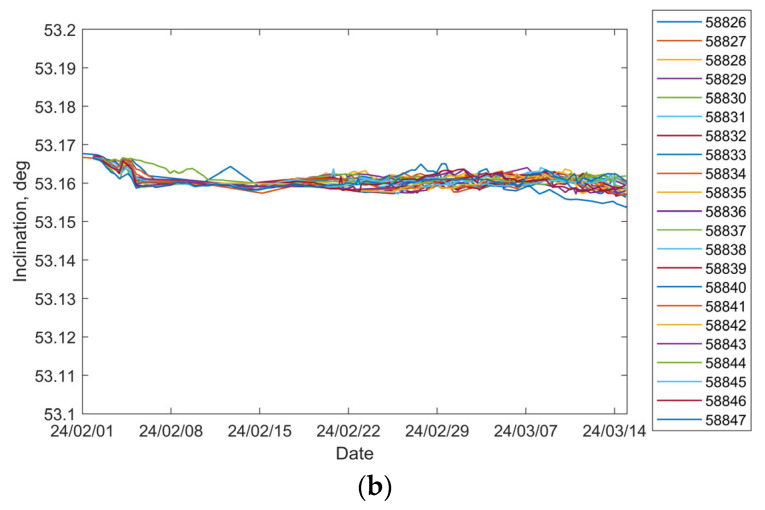
Variations in orbit altitudes and inclinations over time for the chosen batch of Starlink satellites during the orbit-raising phase: (**a**) evolution of orbit altitudes; (**b**) evolution of inclinations.

To investigate the orbital characteristics of Starlink satellites during the orbit-raising phase, a representative set of 22 satellites from the same launch on 24 January 2024 was selected from the TLE catalog. The variations in their orbit altitudes and inclinations with time during the orbit-raising process are shown in Figure 1. Among the selected satellites, 18 experienced two distinct orbital parking stages—approximately at 350 km and 430 km—before reaching their final operational altitude near 480 km. The duration of each parking phase varied among the satellites. In contrast, four satellites—identified by NORAD IDs 58837, 58844, 58845, and 58847—followed a different orbit-raising pattern. These satellites exhibited parking phases at approximately 430 km and 450 km before transitioning to their final orbital altitudes near 480 km and 550 km, respectively. Further analysis of historical TLE data from multiple Starlink batches indicates that, during continuous orbit-raising phases, satellite altitudes tend to increase approximately linearly over time, although at varying rates of ascent. This trend is consistent with the patterns observed in Figure 1a. Given the unknown timing of thruster activation and deactivation, along with the quasi-linear altitude increase observed over multi-day periods, the propulsion system is modeled as delivering a time-averaged, constant thrust acceleration. As illustrated in Figure 1b, the inclination variation among all 22 satellites remains within 0.01°, suggesting that out-of-plane thrust components are negligible during the orbit-raising phase. Moreover, theoretical thrust optimization shows that the efficiency of altitude adjustment is maximized by the along-track thrust. Based on these considerations, the adopted dynamics model assumes a constant equivalent thrust acceleration in the along-track direction.

For LEO constellations like Starlink, the primary perturbative forces arise from the Earth’s non-spherical gravitational field and atmospheric drag. Among these, atmospheric drag is the dominant factor causing semi-major axis decay, offsetting the orbit-raising effect of the along-track thrust. Considering this dynamic interplay, this study incorporates both atmospheric drag and along-track thrust into a unified estimation framework. Under this formulation, the dynamics model is simplified to three primary components: central gravitational acceleration, perturbations due to the Earth’s non-spherical gravitational field, and a constant equivalent low-thrust acceleration, as defined in Equation (4). The perturbation due to the Earth’s non-sphericity $a_{\mathrm{NS}}$ is calculated using the EGM96S geopotential model, truncated at degree and order 30. The combined effect of thrust and atmospheric drag is modeled as a net equivalent acceleration in the along-track direction. Most of the numerical simulations in this study (Section 3.2.1, Section 3.2.2 and Section 3.2.3) are conducted using the simplified model, Equation (4). Only in Section 3.2.4 did we use Equation (21), which extends Equation (4) by incorporating atmospheric drag perturbations.

(4) $$\left\{\begin{array}{l} \frac{dr}{dt}=v\\ \frac{dv}{dt}=-\frac{\mu }{r^{3}}r+a_{\mathrm{NS}}+M_{RTN\to ECI}\left[\begin{array}{l} \;0\\ a_{T}\\ \;0 \end{array}\right] \end{array}\right.$$

### 2.2. Orbit Estimation with an Unscented Batch Filtering Method

Building on the work of Park et al. [30], the study introduces a novel orbit estimation approach for continuously maneuvering, low-thrust satellites by combining the UT with a batch processing technique. Compared to the original formulation, the proposed method adopts an alternative strategy for sigma points sampling that enhances computational efficiency while preserving the estimation accuracy of the UT framework. In addition, it utilizes a simplified dynamics model that explicitly accounts for thrust, thereby improving the robustness and precision of orbit determination under maneuvering conditions. The remainder of this section first presents the sigma points sampling strategy used in this study, followed by a detailed explanation of the integrated “UT-Batch” OD framework.

#### 2.2.1. Sigma Points Sampling Strategy

Symmetric sampling is a widely used strategy in UT-based algorithms, where 2*n* + 1 sigma points are generated to approximate the probability distribution of an *n*-dimensional state vector. However, as the state dimension *n* increases, the computational cost rises sharply. Therefore, optimizing the sigma-point selection strategy becomes crucial, aiming to reduce the number of sigma points while maintaining the accuracy of the distribution approximation. Julier and Uhlmann [32] showed that representing the posterior distribution of an *n*-dimensional state distribution vector requires at least *n* + 1 sigma points. Menegaz et al. [33] conducted a comparative analysis on the performance of UT and UKF using various sigma point sets, including the symmetric set, the rho-minimum sigma set, and a new minimum sigma set. Considering the trade-offs between computational cost, UT performance, and the complexity of parameter tuning, this study adopts the rho-minimum sigma set introduced by Menegaz et al. [31] as the preferred sampling strategy. For clarity and reference, the construction of the *n* + 1 sigma points for an *n*-dimensional state vector with mean $\bar{X}$ and the covariance matrix **P**_xx_ > 0 is presented in the following:

1. Choose the tuning parameter *ω*_0_ such that 0 < *ω*_0_ < 1;
2. Determine the weight matrix ***W*** with *ω*_i_, i = 1, …, n

$$\rho =\sqrt{\frac{1-\omega_{0}}{n}},$$

$$C=\sqrt{I_{n}-\rho^{2}{\left[1\right]}_{n\times n}},$$

(5) $$\omega_{i}={\left(\omega_{0}\rho^{2}C^{-1}{\left[1\right]}_{n\times n}{\left(C^{T}\right)}^{-1}\right)}_{ii},\mathrm{for}\;i=1,\ldots ,n\;,$$

(6) $$W=\left[\begin{array}{l} \omega_{1}\;0\;\;0\\ 0\;\ddots \;\;\;\;0\\ 0\;\;0\;\omega_{n} \end{array}\right].$$

3. Construct the sigma set

(7) $$\left[\chi_{0}\;\;\ldots \;\;\chi_{n}\right]=\left[-\sqrt{P_{xx}}\frac{{\left[\rho \right]}_{n\times 1}}{\sqrt{\omega_{0}}}\;\sqrt{P_{xx}}C{\left(\sqrt{W}\right)}^{-1}\right]+{\left[\bar{x}\right]}_{1:n+1}.$$

The square root of a matrix is calculated through eigenvalue decomposition.

#### 2.2.2. Orbit Estimation Based on an Unscented Batch Filtering Method

The nonlinear system considered in this study consists of the nonlinear dynamics function $f\left(\cdot \right)$ and the measurement function $h\left(\cdot \right)$, formulated as follows:

(8) $$\left\{\begin{array}{l} x_{j+1}=f\left(x_{j},t_{j}\right)\\ z_{j}=h\left(x_{j},t_{j}\right)+\nu_{j} \end{array}\right.$$

where **x**_j_ denotes the state vector at time *t*_j_ with a covariance **P***_j_*, and **z***_j_* represents the measurement vector. The additional measurement noise vector, ***ν****_j_*, is modeled as a zero-mean Gaussian random vector with a covariance of **R***_j_*. Notably, no process noise is added to the system model, as the iterative batch filtering scheme employed in this work ensures convergence through successive refinements. To account for the effects of continuous low-thrust maneuvers, the state vector is augmented beyond the traditional position and velocity vectors in the ECI frame to include the unknown thrust acceleration vector expressed in the RTN frame. Specifically, only the along-track component, denoted as *a*_T_, is estimated, in accordance with the model assumptions described in Equation (4). The measurement vector consists of the slant range *ρ*, radar azimuth *Az* and elevation *El*, **Z** = [*ρ Az El*], all of which are functions of the satellite’s position relative to a ground tracking station. The theoretical calculations of the observations *ρ*, *Az*, and *El* at specific epochs, based on the satellite’s ECI position vector and the geographic coordinates of the ground station, are integrated into the function $h=\left(\cdot \right)$. For detailed derivations and formulations of these observation equations, readers are referred to the relevant literature, such as ref. [3].

Given a sequence of observations $\tilde{Z}$ over the time series *t_j_* for *j* = 1, …, *N*, where *N* denotes the total number of observation epochs, the objective is to estimate the augmented state vector at a selected time *t*_k_, k∈[1,…,N], using both the measurement data and the orbital dynamics model. The rest of this section provides a detailed formulation and implementation of the proposed orbit estimation method based on UT.

The initial estimates of the satellite’s position and velocity, denoted as ***r***_initial_ and ***v***_initial_, are obtained using the Lambert method under the assumption of two-body Keplerian dynamics, with no thrust maneuvers considered. In the absence of prior information regarding the thrust acceleration, its initial value is set to 0 as a general assumption. Accordingly, the initial augmented state estimate ${\hat{x}}_{k}$ and its covariance estimate ${\hat{P}}_{k}$ at the chosen epoch, *t*_k_ is set by

(9) $${\hat{x}}_{k}=[r_{k,\;\mathrm{initial}}\;v_{k,\;\mathrm{initial}}\;0],\;{\hat{P}}_{k}=P_{k,\;\mathrm{initial}},$$

where **P***_k_*_, initial_ is prespecified. The sigma points of ${\hat{x}}_{k}$ are constructed in the way presented in Section 2.2.1, and are denoted by ${\hat{\chi }}_{i,k}$, *i* = 0, …, n.

The sigma set ${\hat{\chi }}_{i,k}$ is then propagated to all measurement moments *t_j_* (*j* = 1, …, *N*, *j* ≠ *k*) using the nonlinear function **f** from Equation (4)

(10) $${\bar{\chi }}_{i,j}=f\left({\hat{\chi }}_{i,k},\;\;t_{j}-t_{k}\right),\;j=1,\ldots ,N\;\mathrm{and}\;j\neq k$$

where ${\bar{\chi }}_{i,j}$ represents the propagated sigma point. At the chosen epoch *t*_k_, the propagated state, covariance, and sigma sets are directly given by their corresponding estimated values

(11) $${\bar{x}}_{k}={\hat{x}}_{k},\;{\bar{P}}_{k}={\hat{P}}_{k},\;{\bar{\chi }}_{i,k}={\hat{\chi }}_{i,k}.$$

At each observation epoch *t_j_*, the theoretical measurements $\gamma_{i,j}$ corresponding to each propagated sigma point are first computed through the nonlinear function **h**, as follows:

(12) $$\gamma_{i,j}=h\left({\bar{\chi }}_{i,j},t_{j}\right),\;i=0,\ldots ,n;\;j=1,\ldots ,N\;\mathrm{and}\;j\neq k.$$

The predicted measurement vector ${\bar{z}}_{j}$ at each observation epoch *t_j_* is then obtained as a weighted sum of the values of $\gamma_{i,j}$ for all propagated sigma points at the corresponding epoch:

(13) $${\bar{z}}_{j}=\sum_{i=0}^{n}\omega_{i}\gamma_{i,j},\;j=1,\ldots ,N\;\mathrm{and}\;j\neq k.$$

Given that both $\gamma_{i,j}$ and ${\bar{z}}_{j}$ originate from the state at epoch *t*_k_, the sets consisting of $\gamma_{i,j}$ and ${\bar{z}}_{j}$ for all measurement moments (with the exception of the *t*_k_ moment) are denoted by ${\tilde{\gamma }}_{i,k}$ and ${\bar{Z}}_{k}$, respectively,

(14) $${\tilde{\gamma }}_{i,k}=\left[\begin{array}{l} \gamma_{i,1}\\ \gamma_{i,2}\\ \;\;\vdots \\ \gamma_{i,N} \end{array}\right],\;{\bar{Z}}_{k}=\left[\begin{array}{l} \;{\bar{z}}_{1}\\ \;{\bar{z}}_{2}\\ \;\;\vdots \\ \;{\bar{z}}_{N} \end{array}\right].$$

Then, the covariances for the propagated measurements ${\bar{P}}_{k}^{Z}$ can be derived as follows:

(15) $${\bar{P}}_{k}^{Z}=\sum_{i=0}^{n}\omega_{i}\left({\tilde{\gamma }}_{i,k}-{\bar{Z}}_{k}\right){\left({\tilde{\gamma }}_{i,k}-{\bar{Z}}_{k}\right)}^{T}+{\tilde{R}}_{k}$$

where ${\tilde{R}}_{k}$ is the measurement noise matrix and the cross-correlation matrix ${\bar{P}}_{k}^{xZ}$ is given as follows:

(16) $${\bar{P}}_{k}^{xZ}=\sum_{i=0}^{n}\omega_{i}\left({\bar{\chi }}_{i,k}-{\bar{x}}_{k}\right){\left({\tilde{\gamma }}_{i,k}-{\bar{Z}}_{k}\right)}^{T}.$$

Furthermore, the filter gain **K***_k_* is calculated according to the following expression:

(17) $$K_{k}={\bar{P}}_{k}^{xZ}{\left({\bar{P}}_{k}^{Z}\right)}^{-1}.$$

The measurement residual is determined as the difference between the actual measurement ($\tilde{Z}$) and the predicted measurement (${\bar{Z}}_{k}$) at all measurement moments

(18) $$\Delta {\tilde{z}}_{k}=\tilde{Z}-{\bar{Z}}_{k}.$$

The satellite augmented state vector at the selected epoch *t*_k_ is updated as indicated below

(19) $${\hat{x}}_{k}={\bar{x}}_{k}+K_{k}\Delta {\tilde{z}}_{k}.$$

An iterative procedure is applied to estimate the satellite’s augmented state vector ${\hat{x}}_{k}$. The process continues until the maximum number of iterations is reached, or the absolute value of the difference between the root mean square (RMS) values of the measurement residuals from two consecutive iterations is smaller than a predefined convergence criterion, designated as *ε*

(20) $${\mathrm{RMS}}_{\mathrm{new}}=\frac{\Delta {\tilde{z}}_{k}^{T}{{\tilde{R}}_{k}}^{-1}\Delta {\tilde{z}}_{k}}{N},\;\left|\frac{{\mathrm{RMS}}_{\mathrm{new}}-{\mathrm{RMS}}_{\mathrm{old}}}{{\mathrm{RMS}}_{\mathrm{old}}}\right|<\varepsilon .$$

**Figure 2:**
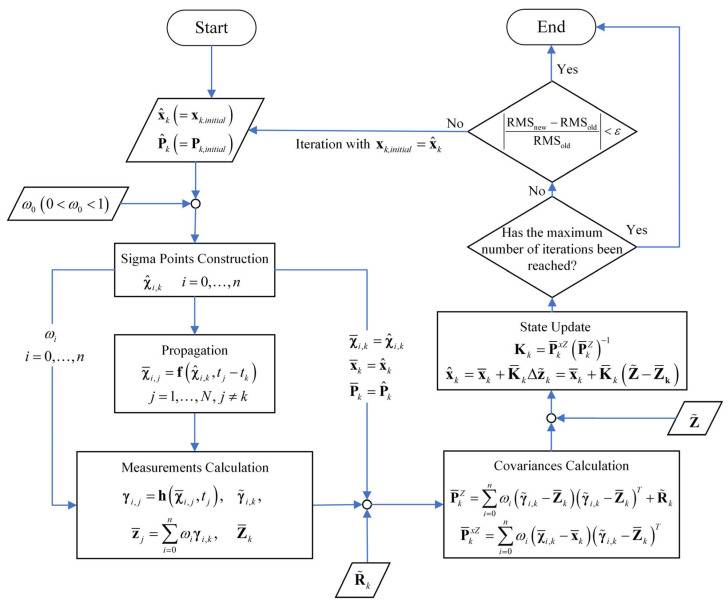
The flowchart of the OD procedure based on an unscented batch filtering method.

Since the OD approach presented in this section adopts a batch processing scheme to estimate the satellite’s augmented state vector at a specific epoch, the iterative procedure does not involve propagating state covariances to other measurement epochs. As a result, this method avoids potential issues commonly encountered in sequential estimators, such as instability due to covariance propagation. Upon the completion of each iteration, the initial state covariance is reinitialized. The flowchart of the OD procedure based on an unscented batch filtering method is shown in Figure 2.

## 3. Simulations and Results

In this section, the methodology introduced in Section 2 is applied to estimate the orbits of continuously ascending Starlink satellites, as well as their equivalent thrust acceleration. Following the estimation results, OP is carried out. The reference orbits, hereafter referred to as the “true orbits”, are constructed from ephemerides published by SpaceX on the Space-Track.org website. These orbits serve as the ground truth for both generating simulated observations and quantitatively evaluating the accuracy of OD and OP. Each SpaceX ephemeris file contains position and velocity data over a three-day period along with associated covariance matrices. These files are updated three times daily at 8 h intervals. To ensure accuracy, the reference orbit is constructed by merging the initial 8 h segments from each ephemeris file, as these segments are characterized by lower uncertainty according to the provided covariance matrices. Simulated radar observations are generated based on the “true orbits”, ground station coordinates, predefined observation errors, and operational constraints such as range and elevation limits. State propagation is carried out using the reduced dynamics model defined in Equation (4), which incorporates the constant low-thrust acceleration described in Section 2.

### 3.1. Observation Simulations

To analyze the spatial and temporal distribution characteristics of ground-based radar observations for continuously ascending Starlink satellites, observation simulations are carried out involving 22 satellites of the V2.0 Mini version (NORAD IDs: 58826–58847) launched on 24 January 2024. Observations are simulated from three different ground station locations selected to provide diverse geometric visibility conditions. The geographic coordinates of the station sites used in this study are shown in Table 1. The measurement data is simulated at 30-s intervals over a 7-day period, starting from 22:32:42 UTC on 7 February 2024.

Theoretical observations (*ρ*, *Az*, *El*) are obtained by first calculating the satellite’s position relative to the radar station in the Earth-Centered, Earth-Fixed (ECEF) coordinate system. This relative position is then transformed into the local East-North-Up (ENU) frame of the ground station, where the x-axis points east, the y-axis points north, and the z-axis aligns with the local zenith. To simulate radar measurements, observational errors are introduced, with standard deviations of 100 m for range *ρ* and 100 arcseconds for both azimuth *Az* and elevation *El*. Observable arcs over the 7-day period are determined by applying the radar’s operational constraints: a maximum range of 2000 km (*ρ* ≤ 2000 km) and a minimum elevation angle of 5° (*El* ≥ 5°).

**Figure 3:**
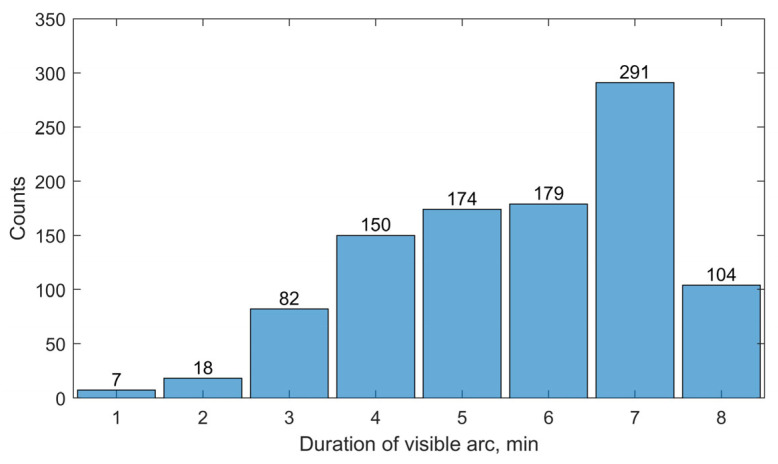
Visible arc statistics for the targeted satellites, obtained by Station 1 during the observation period he flowchart of the OD procedure based on an unscented batch filtering method.

**Figure 4:**
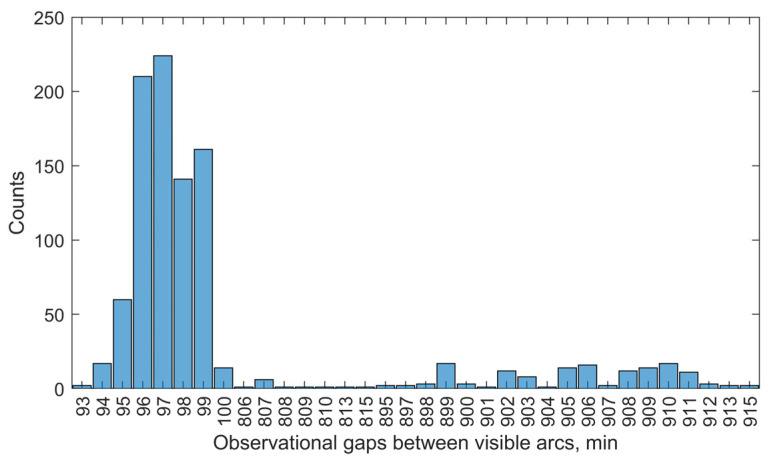
Statistics of observational gaps obtained by Station 1 during the observation period.

**Figure 5:**
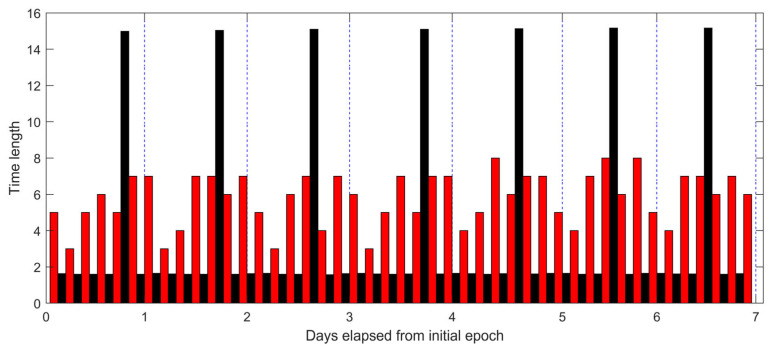
Distribution of visibility arcs for Starlink satellite with NORAD ID 58836, recorded by Station 1 during the observation period. The red columns indicate durations of the visible arcs, which are measured in minutes. The black columns represent durations of the observational gaps, which are measured in hours.

A total of 1005 visible arcs were recorded for the 22 target satellites over a 7-day observation period using Station 1. Figure 3 illustrates the statistical distribution of visible arc durations, measured in minutes. As shown, the majority of the arcs (89%) fall within the 4–8-min range, with the highest frequency observed at 7 min (291 arcs). Figure 4 presents the distribution of observational gaps, defined as the time intervals between consecutive visible arcs. The results show that 84% of these gaps have a duration of approximately 1.5 h (93–100 min), while most of the remaining gaps last around 15 h (806–915 min). Figure 5 displays the distribution of visible arcs for the Starlink satellite with NORAD ID 58836 during the same observation period. The durations of the visible arcs, shown in red, are measured in minutes, whereas the observational gaps, shown in black, are indicated in hours. On average, 6–7 visible arcs are obtained per day, totaling approximately 40 min of daily visibility. Notably, each day’s observational gaps invariably include a prolonged interval lasting about 15 h, with shorter gaps interspersed throughout the remaining period.

In addition to Station 1, Station 2 and Station 3 were chosen to simulate observations of the same set of target satellites over the identical 7-day period. A total of 529 visible arcs were obtained from Station 2, and 957 arcs were recorded from Station 3. For Station 2, each satellite had an average of 3–4 access opportunities per day, with a total daily visibility of about 20 min. A distinct observational gap lasting 11–13 h is consistently recorded each day. Owing to the comparable latitude coordinates between the station sites, the statistical characteristics derived from Station 3 demonstrated substantial alignment with those obtained from Station 1. Specifically, Station 3 provided an average of 6–7 visible arcs per satellite per day, with a cumulative daily visibility of approximately 40 min. Also, a prolonged observational gap lasting about 15 h is consistently identified within each daily cycle. These findings suggest that, when initiating observations from randomly selected epochs, an observation window exceeding 24 h is recommended to ensure sufficient access coverage and to support robust OD performance.

### 3.2. Orbit Determination Simulation

**Figure 6:**
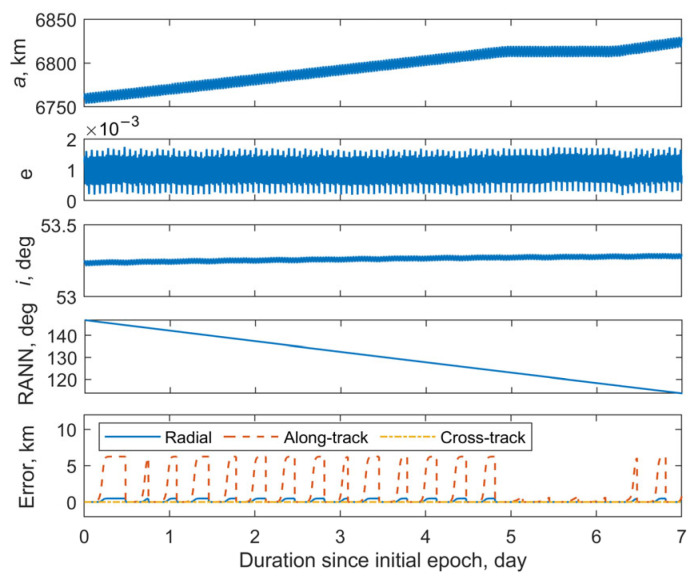
Reference orbit and position errors of Starlink satellite with the NORAD ID 58831 since epoch 7 February 2024 19:25:42 UTC.

This section examines the orbit of the Starlink satellite with NORAD ID 58831. Figure 6 shows the reference orbit and corresponding position errors, derived from the SpaceX ephemeris, over a seven-day period starting from the epoch 7 February 2024 19:25:42 UTC. It can be seen that during the first 5 days, the semi-major axis increases almost linearly at a rate of approximately 10.8 km/day. On the sixth day, it remains nearly constant, and from the seventh day onwards, the linear growth pattern resumes. During the period of linear growth of the semi-major axis, the position error can reach approximately 6.5 km. In contrast, when the semi-major axis remains constant, the position error is less than 1 km. The eccentricity and the inclination during the 7 days remain nearly constant. Simulated observations with Station 1 were conducted over a 24 h period beginning at the same epoch, following the procedure described in Section 3.1. In total, 76 sets of observational data were obtained, resulting in seven visible arcs (see Table 2). The time intervals between the consecutive visible arcs were approximately 1.5 h. The first observable moment, i.e., 7 February 2024 20:31:42 UTC, is taken as the selected epoch *t*_k_ for orbit determination. At this epoch, the true position and velocity vectors are $r_{0}^{true}$ = [−5956286.9372 1295514.0729 2914748.1930] m and $v_{0}^{true}$ = [1347.5254 −5509.4750 5184.0691] m/s. The initial estimation of the satellite’s position and velocity (***r***_initial_, ***v***_initial_) at the selected epoch *t*_k_ is computed using Lambert’s method, based on the inertial positions corresponding to the first and last timestamps of Arc No. 1, derived from the observational data. The tuning parameter *ω*_0_ for sigma points construction is set to 0.3. The predefined convergence criteria *ε* are taken as 10^−2^. The maximum number of iterations is set to 30.

As discussed in Section 2, the proposed method removes the requirement for process noise. However, the covariance matrices associated with the initial state vector (**P**_initial_) and the measurement noise (**R**) must still be empirically specified. Multiple combinations of **P**_initial_ and **R** are tested to evaluate their influence on OD performance. Once the augmented state vector at the chosen epoch is determined, they are propagated through the dynamics equations to generate orbit ephemerides over the following 24 h period. To evaluate the accuracy of OD and OP, RMS errors between the generated orbit and the true orbit are calculated over the next 24 h in the radial, along-track, cross-track, and total position components.

#### 3.2.1. Effects of the Measurement Errors

In this study, the impact of measurement errors is reflected in two main aspects. First, the simulated observations are generated by intentionally introducing measurement errors. Second, this noise is used to calculate the covariances in Equation (15), which subsequently affects the performance of the filter.

**Figure 7:**
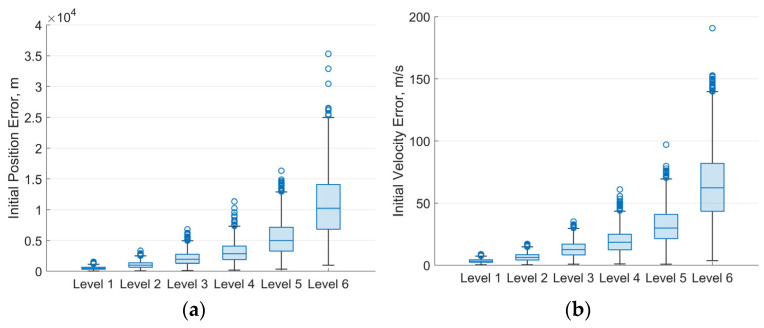
Box chart of initial state errors caused by different levels of measurement noise: (**a**) Box chart of initial position error; (**b**) Box chart of initial velocity error.

The magnitude of initial state errors caused by different levels of measurement noise is estimated first. Measurement noise is applied at seven levels. The standard deviations for range *ρ*, azimuth *Az*, and elevation *El* are set as follows: [50 m, 50 arcsec, 50 arcsec] for Level-1; [100 m, 100 arcsec, 100 arcsec] for Level-2; [200 m, 200 arcsec, 200 arcsec] for Level-3; [300 m, 300 arcsec, 300 arcsec] for Level-4; [500 m, 500 arcsec, 500 arcsec] for Level-5; and [1000 m, 1000 arcsec, 1000 arcsec] for Level-6. For every case, the Monte Carlo method is applied to analyze the distribution of errors between the estimated initial states and the true orbital value. Figure 7 shows the distribution of initial state estimation errors for 1000 simulation runs of IOD under various measurement noise levels. A clear upward trend in the median, upper, and lower quartiles can be observed as the noise level increases. This indicates that higher measurement noise not only leads to less accurate orbit initialization but also introduces greater uncertainty in the estimates.

Taking into account the capabilities of modern observation technology, as well as potential differences in observational accuracy among various devices, the measurement noise of Levels 2–4 was used to simulate 3 sets of pseudo-radar observations. The initial state errors (deviations of (***r***_initial_, ***v***_initial_) from ($r_{0}^{true}$, $v_{0}^{true}$)) calculated values by each set of simulated observations are as follows:

- Case 1: Δ***r***_0_ = [−195.4272, 915.1713, −364.8435] m, Δ***v***_0_ = [0.4716, −3.7691, −1.9408];
- Case 2: Δ***r***_0_ = [1295.5440 −863.3109 −1545.5358] m, Δ***v***_0_ = [−6.1658 3.6929 6.2951];
- Case 3: Δ***r***_0_ = [585.9460 2818.3729 −1410.7564] m, Δ***v***_0_ = [2.6370 −14.4218 −18.6149].

The OD algorithm was tested using numerical analysis with four different levels of measurement noise for each case: [50 m, 50 arcsec, 50 arcsec], [100 m, 100 arcsec, 100 arcsec], [300 m, 300 arcsec, 300 arcsec], and [1000 m, 1000 arcsec, 1000 arcsec]. In all tests, covariance values for the initial position and velocity uncertainties are set to [(10,000 m)^2^, (10,000 m)^2^, (10,000 m)^2^, (10 m/s)^2^, (10 m/s)^2^, (10 m/s)^2^]. As suggested in [27], for LEO mega-constellations equipped with low-thrust electric propulsion systems, the low-thrust often has a magnitude of *J*_2_^2^. Based on their use of a prior thrust error, the initial covariance for the along-track acceleration component *a*_T_ is assumed to be (10^−4^ m/s^2^)^2^.

**Figure 8:**
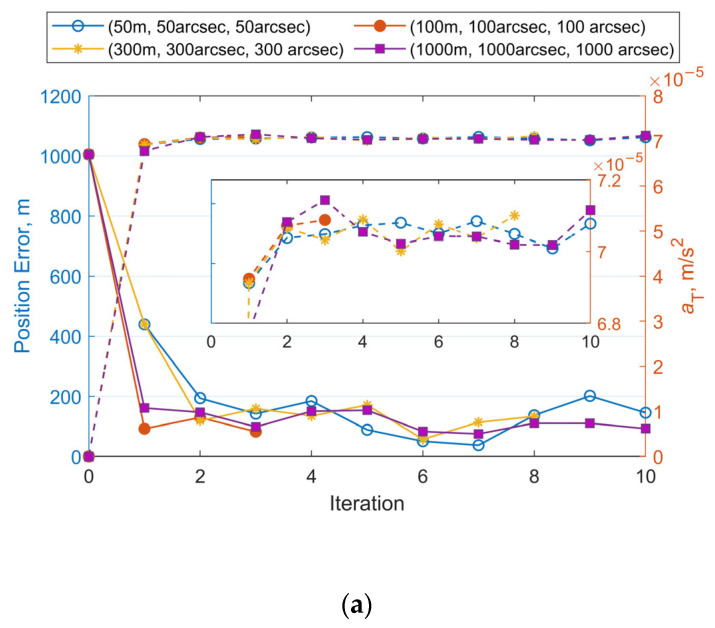

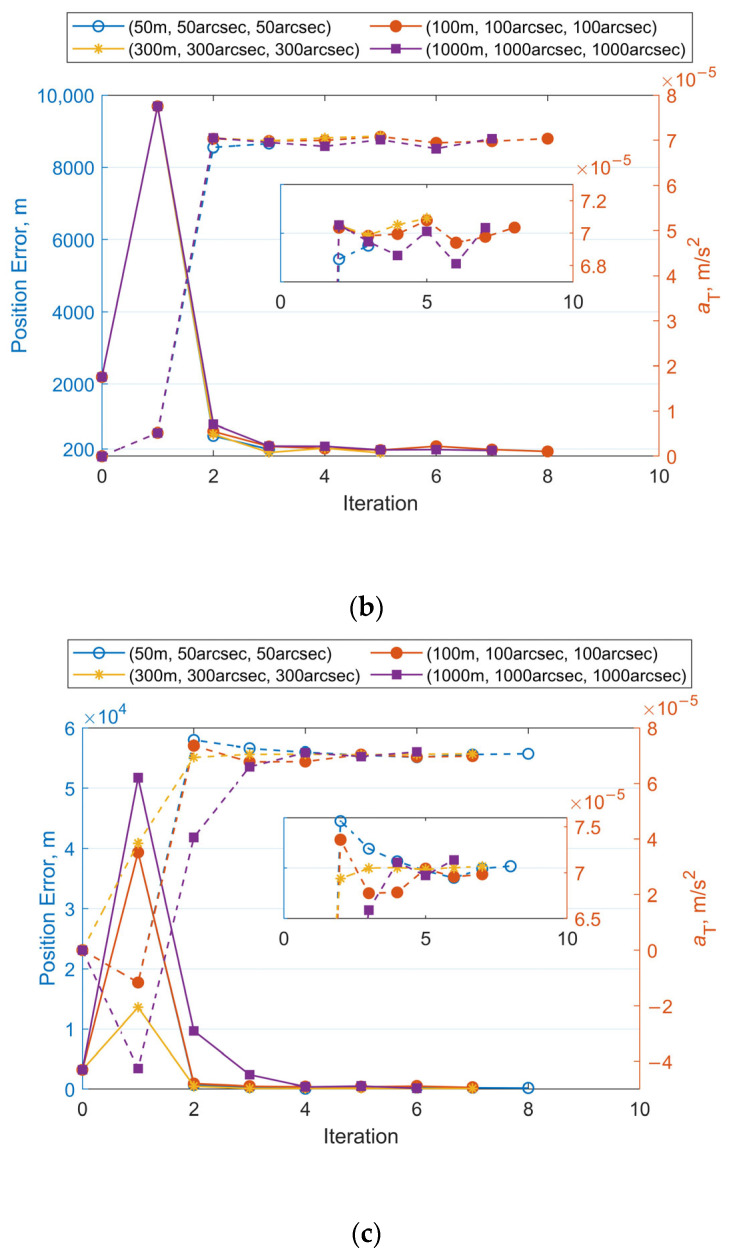
Iterative histories of position errors and *a*_T_ estimates at the chosen epoch with varying measurement noise levels for OD: (**a**) Position errors and a_T_ estimates vs. iteration number for Case 1. (**b**) Position errors and *a*_T_ estimates vs. iteration number for Case 2. (**c**) Position errors and a_T_ estimates vs. iteration number for Case 3.

**Figure 9:**
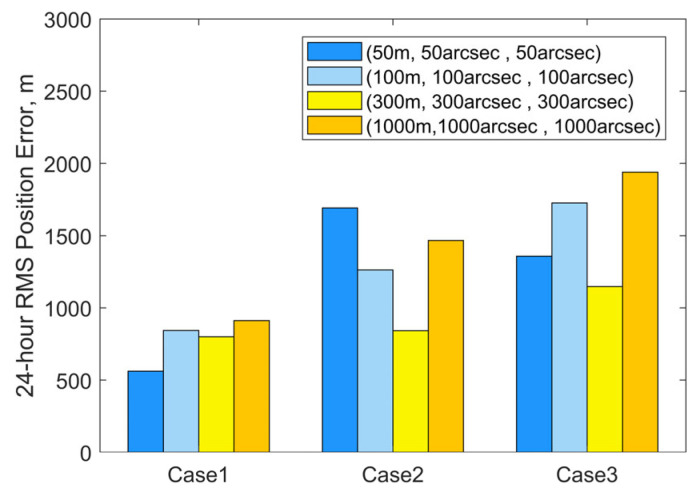
24 h RMS three-dimensional position errors based on OD results for varying measurement noise levels.

Figure 8a–c illustrate the iterative histories of position errors and along-track thrust acceleration (*a*_T_) estimates at the chosen epoch for Cases 1–3, respectively. The solid lines with different markers represent the position errors, while the dashed lines with corresponding markers denote the estimated values of *a*_T_. Each color is associated with a specific measurement noise level. It can be seen that all numerical test cases meet the convergence criterion within 10 iterations. Using the same set of observational data (with identical initial errors), the final position errors and acceleration estimates converge to similar values after the iterations, even when different measurement noise levels are applied during orbit determination. At the selected epoch, the mean position error is 114 m and the estimated mean value of *a*_T_ is 7.095 × 10^−5^ m/s^2^ for Case 1. For Case 2, these values are 163 m and 7.058 × 10^−5^ m/s^2^. For Case 3, the results are 200 m and 7.065 × 10^−5^ m/s^2^. As the accuracy of the observational data decreases from Case 1 to Case 3, more iterations are needed to reach the final result. The position errors at the selected epoch also increase. Case 1 achieves position errors below 200 m by the second iteration. Case 2 requires until the third iteration to reduce errors below 300 m, while Case 3 needs three to four iterations to reach sub-500 m precision. Orbit propagation was then performed using the OD results (position, velocity, and the along-track acceleration) from these tests and Equation (4). Figure 9 summarizes the RMS three-dimensional position errors of the predicted orbits over 24 h. Overall, the 24 h RMS position errors for all cases remain below 2000 m. The more accurate the observational data, the smaller the errors in orbit determination and prediction. For the same observational data, the accuracy of OD and OP is nonlinearly related to the measurement noise used in the OD process. Additionally, it is observed that smaller OP position errors occur when the measurement noise level used for observational data generation matches that employed in the OD process.

#### 3.2.2. Effects of the Initial State Covariance

This subsection is dedicated to the examination of the impact of varying initial state covariances. Two sets of observational data from Case 1 and Case 3 in Section 3.2.1 are used in this subsection for comparison. For each set of observational data, six test cases are defined, each with progressively increasing initial covariance values for position and velocity components. The corresponding diagonal elements of the initial covariance matrix **P**_initial_ are set as follows:

- Case A: (100 m)^2^ for position, (0.1 m/s)^2^ for velocity;
- Case B: (1000 m)^2^ for position, (1 m/s)^2^ for velocity;
- Case C: (5000 m)^2^ for position, (5 m/s)^2^ for velocity;
- Case D: (10,000 m)^2^ for position, (10 m/s)^2^ for velocity;
- Case E: (50,000 m)^2^ for position, (50 m/s)^2^ for velocity;
- Case F: (100,000 m)^2^ for position, (100 m/s)^2^ for velocity.

These values are assigned without reference to the statistical results shown in Figure 7, as prior knowledge may not be available for most practical cases. In all cases, the measurement noise level is set to be [300 m, 300 arcsec, 300 arcsec], the initial covariance for the along-track acceleration component *a*_T_ is assumed to be (10^−4^ m/s^2^)^2^.

**Figure 10:**
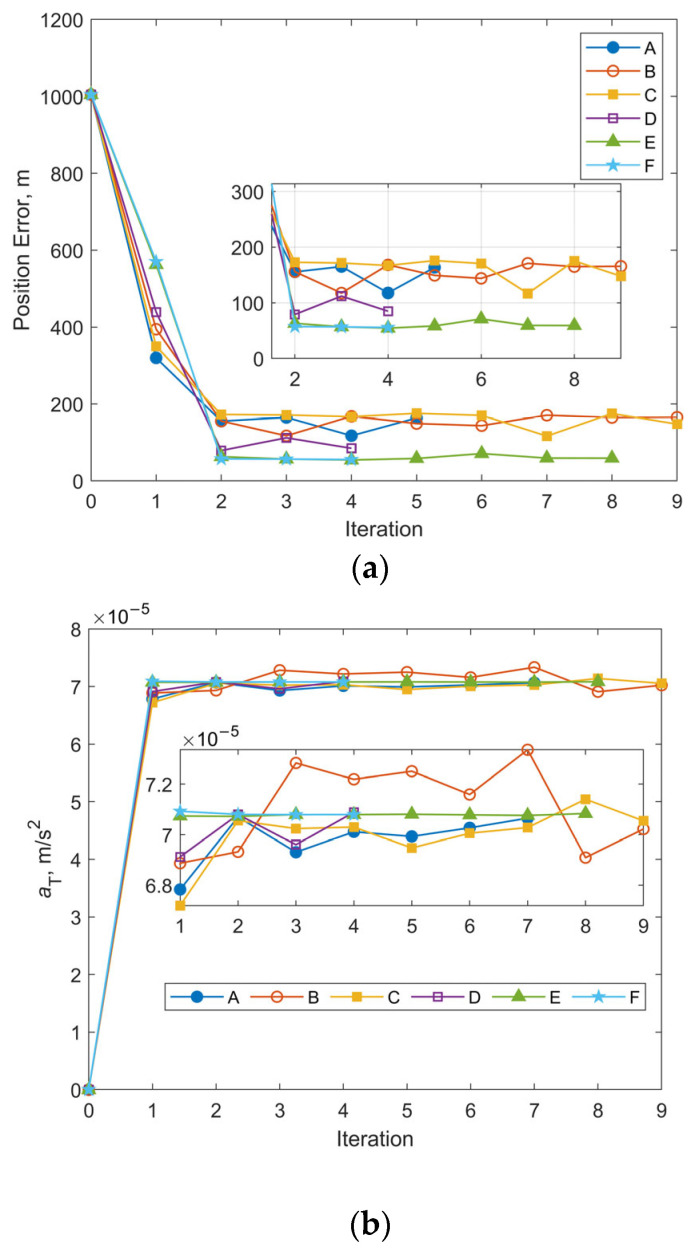
Iterative histories of position errors and *a*_T_ estimates at the chosen epoch for Case 1 with different initial covariance values of position and velocity: (**a**) position errors vs. iteration number for Case 1; (**b**) *a*_T_ estimates vs. iteration number for Case 1.

**Figure 11:**
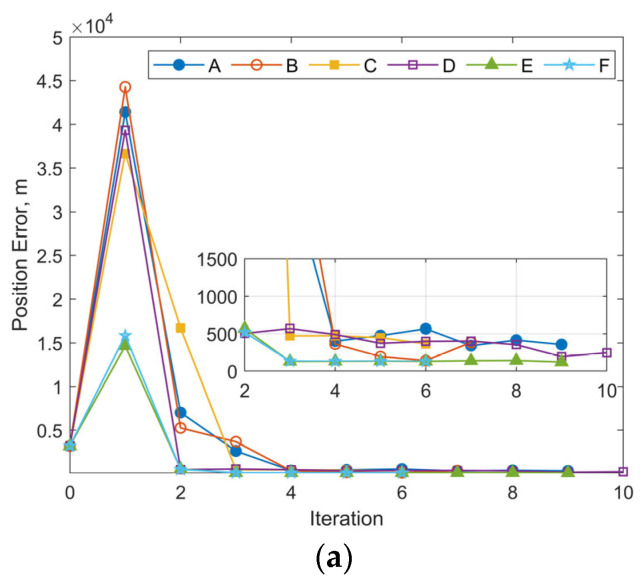

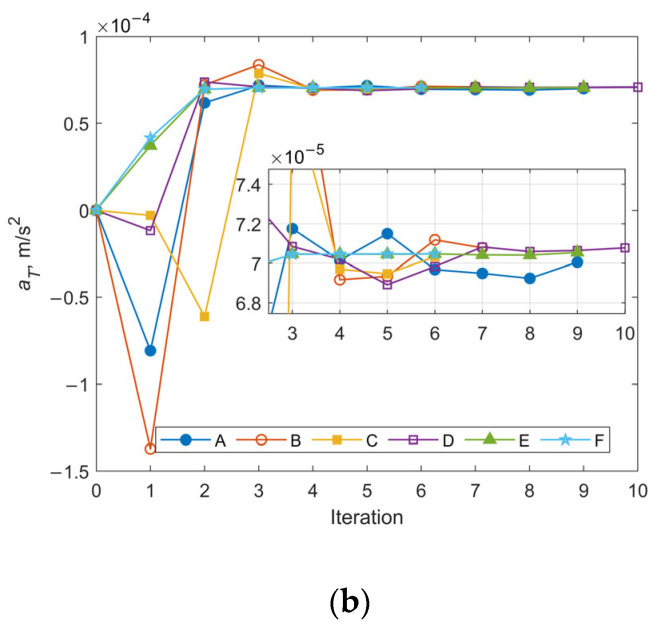
Iterative histories of position errors and *a*_T_ estimates at the chosen epoch for Case 3 with different initial covariance values of position and velocity: (**a**) position errors vs. iteration number for Case 3; (**b**) *a*_T_ estimates vs. iteration number for Case 3.

**Figure 12:**
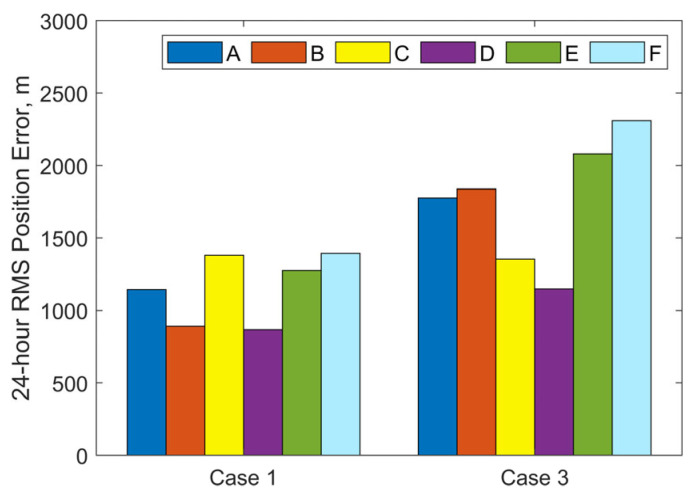
24 h RMS three-dimensional position errors based on OD results for different initial covariance values of position and velocity.

Figure 10 and Figure 11 illustrate the iterative histories of position errors and the along-track thrust acceleration (*a*_T_) estimates at the chosen epoch for Cases 1 and 3 with various initial covariance values of position and velocity. As shown in Figure 10 and Figure 11, all tests for both Case 1 and Case 3 converge within 10 iterations. For Cases A–D, the convergence of both position errors and *a*_T_ estimates exhibits oscillatory behavior near their final values. In contrast, Cases E and F with larger initial covariance values for position and velocity demonstrate significantly improved stability after convergence, along with smaller final position errors. The resulting RMS position errors for 1-day OP are shown in Figure 12. Notably, although Cases E and F yield smaller position and velocity errors following OD at the specified epoch, their RMS position errors during OP are actually larger. The cause is the underestimated uncertainty of equivalent along-track acceleration, which is incompatible with the significantly greater uncertainty of position. Additional numerical tests were performed using an initial covariance of (10^−2^ m/s^2^)^2^ for *a*_T_, while keeping all other parameters consistent with Case 1-E, Case 1-F, Case 3-E, and Case 3-F. These modified configurations are designated as Case 1-E′, Case 1-F′, Case 3-E′, and Case 3-F′, respectively. The positional errors of 24 h OP after OD using these modified and their corresponding original configurations are presented in Table 3. Clear reductions can be observed in both RMS and the maximum position errors using modified configurations. As evidenced by the results, the RMS position error improvements range from a modest 13% reduction (when comparing Case 1-F’ relative to 1-F) to a substantial 49% decrease (observed between Case 3-E’ and Case 3-E). Similarly, maximum position error reductions show comparable enhancement, varying between 22% for Case 1-F’ and 48% for Case 3-E’. These results highlight the significant influence that proper state covariance initialization can have on the accuracy of both OD and OP. It seems that a favorable balance between OD stability and OP accuracy can be achieved by setting the uncertainties of position, velocity, and the equivalent along-track acceleration to several tens of kilometers, tens of meters per second, and the order of 10^−2^ m/s^2^, respectively.

#### 3.2.3. Statistical Analysis with Various Satellites

A comprehensive validation was conducted for all 22 of the Starlink satellites mentioned in Section 3.1, with NORAD IDs 58826–58847. This validation followed a three-phase procedure: (1) observational data simulation, (2) orbit determination, and (3) 24 h orbit prediction. The observation simulation began on 7 February 2024 and lasted 24 h for every satellite. According to Vallado [3], ground-based radar is commonly used to observe LEO satellites. Typical measurement accuracy is several tens of meters for range (*ρ*) and several tens of arcseconds for angles (*Az*, *El*). In this subsection, two measurement noise levels are used for comparison: [100 m, 100 arcsec, 100 arcsec] and [200 m, 200 arcsec, 200 arcsec]. These conservative, higher noise values are chosen to test the performance of our method. If higher-precision measurements are available, the performance of our approach is expected to improve accordingly. The initial state covariance values, representing the uncertainty about the satellite’s Earth-Centered Inertial (ECI) position and velocity vectors as well as the along-track acceleration, were set to [(10,000 m)^2^, (10,000 m)^2^, (10,000 m)^2^, (10 m/s)^2^, (10 m/s)^2^, (10 m/s)^2^, (10^−4^ m/s^2^)^2^]. The simulation results presented in Section 3.2.1 and Section 3.2.2 demonstrate that this configuration of initial state uncertainties achieves stable and favorable orbit determination and prediction performance.

**Figure 13:**
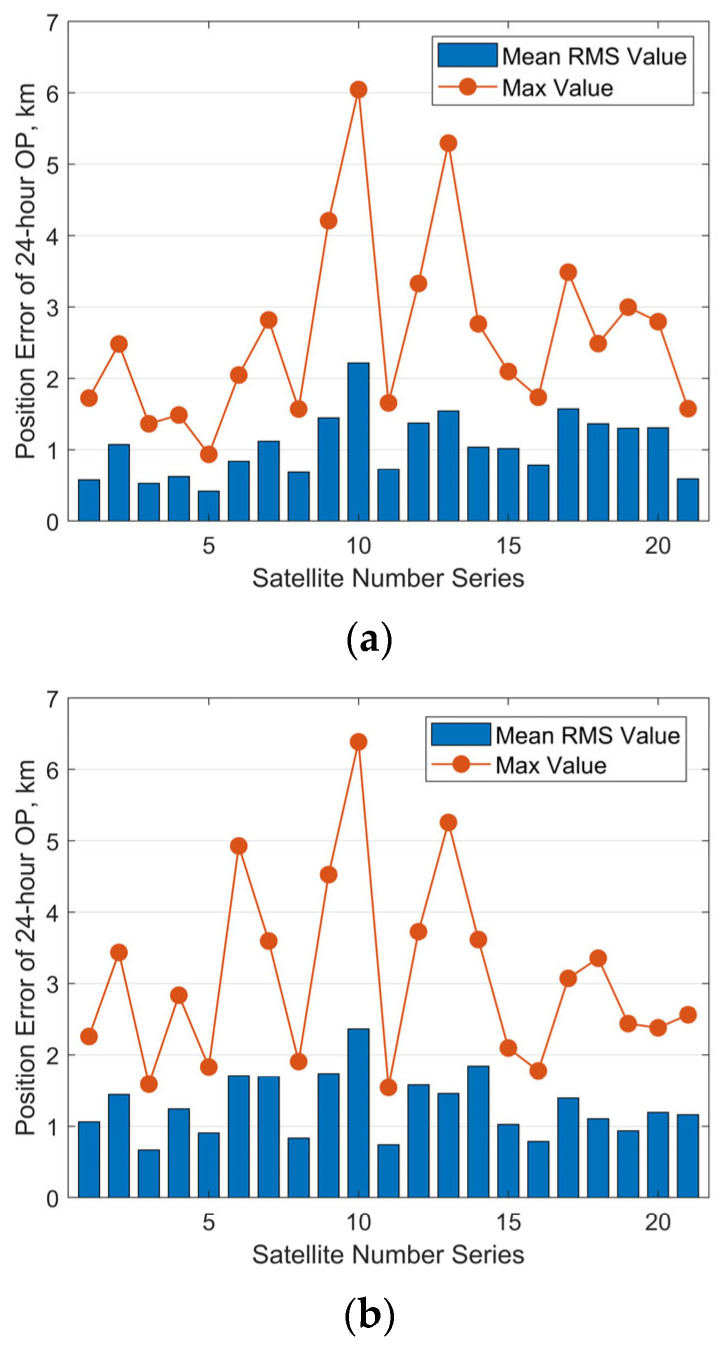
Predicted position errors over 24 h for 21 climbing Starlink satellites: (**a**) with measurement noise level of [100 m, 100 arcsec, 100 arcsec]; (**b**) with measurement noise level of [200 m, 200 arcsec, 200 arcsec].

A statistical analysis was performed by performing 30 independent observation simulations, OD and OP for each satellite. The 24 h RMS position errors of OP were computed for all trials. Figure 13 presents the resulting mean RMS and maximum position errors for each satellite (except the satellite with NORAD ID 58837). For clarity in the graphical representation, the satellite number series on the x-axis corresponds directly to the NORAD IDs in ascending order. The results show that, for a measurement noise level of [100 m, 100 arcsec, 100 arcsec], the maximum position prediction errors are all less than 6 km. The mean RMS values of the position prediction errors are below 2.5 km. For a noise level of [200 m, 200 arcsec, 200 arcsec], the maximum position prediction errors remain below 7 km, and the mean RMS values are still under 2.5 km. In general, decreased observation accuracy results in greater errors in orbit determination and prediction for most satellites. It should be noted that, during the observation simulation period, the observational data for 14 satellites contained a gap of approximately 15 h. The achieved level of OP accuracy demonstrates that our method remains robust and reliable even in the presence of observational gaps of around 10 h.

**Figure 14:**
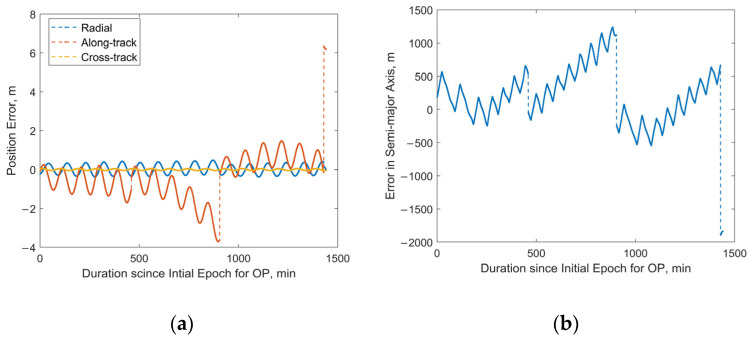
Predicted position and semi-major axis errors over 24 h for Starlink satellite with NORAD ID 58837: (**a**) position errors; (**b**) semi-major axis errors.

**Figure 15:**
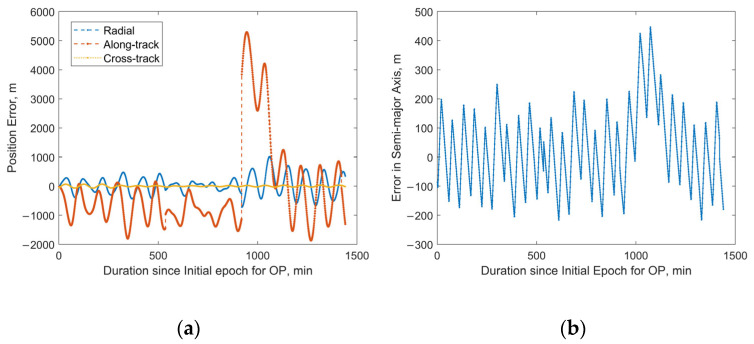
Predicted position and semi-major axis errors over 24 h for Starlink satellite with NORAD ID 58839: (**a**) position errors; (**b**) semi-major axis errors.

**Figure 16:**
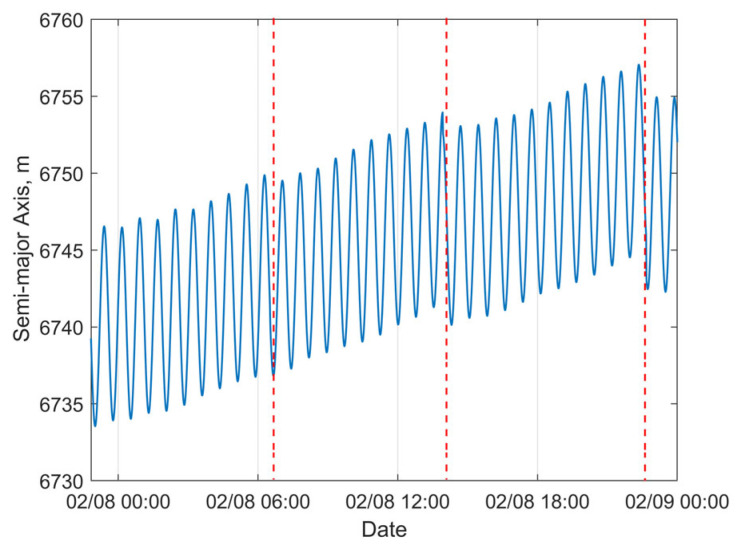
Evolution of “true” semi-major axis for Starlink satellite 58837.

For the satellite with NORAD ID 58837 launched in the same batch, the 24 h predicted RMS position error after OD can reach approximately 13 km, with a maximum value of about 60 km, as shown in Figure 14a. Sudden jumps are observed in the predicted position and semi-major axis errors, which are caused by discontinuities in the “true orbit”. Such discontinuities are systematically present because the “true orbit” is generated through stitching multiple ephemeris files, where discontinuities may occur at file transition epochs. Taking the satellite with NORAD ID 58839 as an example, Figure 15a,b demonstrate that our method achieves robust orbit determination, despite the presence of orbital discontinuities. The 24 h prediction RMS position error is about 1.54 km. However, excessively large jumps may potentially indicate orbital anomalies. For Starlink satellites in the orbit-raising phase, such jumps are typically associated with significant changes in the rate of the semi-major axis (orbital altitude) variation. The variation in the “true” semi-major axis with time during the OP period for the satellite with NORAD ID 58837 is illustrated in Figure 16. The red dashed lines in the figure divide the evolution of the semi-major axis into four segments, each characterized by different rates of semi-major axis change. This change in the orbital behavior of Starlink 58837 around 8 February 2024 is also clearly captured in Figure 1a, where the TLE-derived orbital altitude profiles show significantly different rates of change. Under such circumstances, the constant along-track acceleration assumption employed in our reduced orbital dynamics model becomes invalid, leading to significant OD and OP errors.

#### 3.2.4. Effects of a Higher-Fidelity Dynamics Model

To further evaluate the performance of the proposed OD method based on the reduced dynamics model, a higher-fidelity orbital dynamics model was additionally employed for comparison. The higher-fidelity model explicitly models the perturbation acceleration due to atmospheric drag (***a***_AD_) as a distinct component, separate from the low-thrust acceleration, as defined in Equation (21). The NRLMSISE-00 atmospheric mass density model [34] is used to calculate ***a***_AD_. The area-to-mass ratio and the drag coefficient *C*_d_ are assumed to be 0.02 m^2^/kg and 2.2, respectively. The model and related parameters are used throughout both the OD process and the subsequent OP.

(21) $$\left\{\begin{array}{l} \frac{dr}{dt}=v\\ \frac{dv}{dt}=-\frac{\mu }{r^{3}}r+a_{\mathrm{NS}}+a_{\mathrm{AD}}+M_{RTN\to ECI}\left[\begin{array}{c} 0\\ a_{T}\\ 0 \end{array}\right] \end{array}\right.$$

**Figure 17:**
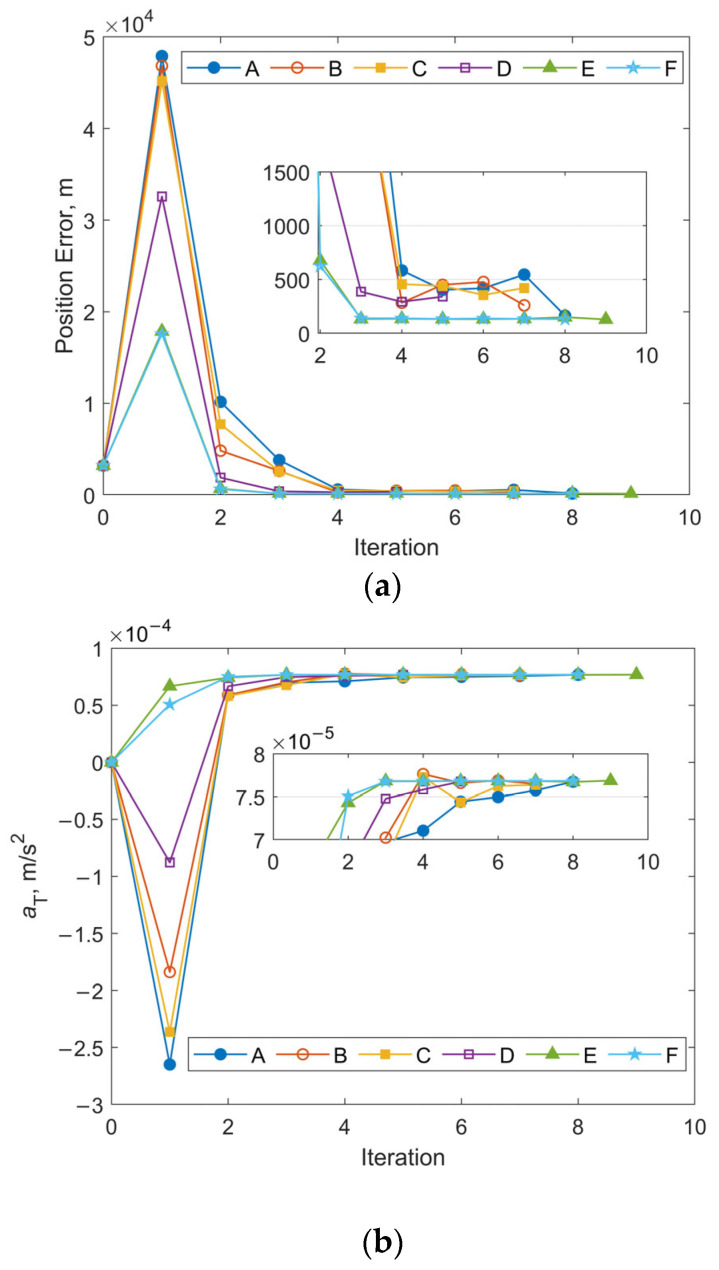
Iterative histories of position errors and *a*_T_ estimates at the chosen epoch obtained through OD using Equation (21): (**a**) position errors vs. iteration number for Case 3 with different initial covariance values of position and velocity; (**b**) *a*_T_ estimates vs. iteration number for Case 3 with different initial covariance values of position and velocity.

Numerical simulations were conducted using the same observational data as Case 3 in Section 3.2.1, while maintaining all other parameters consistent with those specified in Section 3.2.2. This includes the initial covariance values for position, velocity, and along-track acceleration, as well as the measurement noise level. Figure 17a,b illustrate the iteration histories of position errors and *a*_T_ estimates at the selected epoch, respectively. Compared with Figure 11, the overall iterative trend remains consistent. Cases E and F, which adopt larger initial covariance values for position and velocity, demonstrate significantly improved stability as well as smaller final position errors. The magnitude of the position error at the chosen epoch for these 2 cases is also similar to that observed in Figure 11, at approximately 130 m. The average estimated value of the along-track acceleration shown in Figure 17b is about 7.66 × 10^−5^ m/s^2^, and the value for cases shown in Figure 11b is 7.04 × 10^−5^ m/s^2^.

For comparison, the 24 h OP RMS position errors, calculated based on the final OD results using different motion equations, are summarized in Table 4. The corresponding maximum position errors are presented in Table 5. The results show that for cases using the higher-fidelity model with Equation (21), similar to performance observed with Equation (4), although Cases E and F achieve lower positional/velocity errors at the selected epoch after OD, the 24 h RMS and maximum position errors of OP are actually larger. Despite this, for all Cases A–F, adopting the high-fidelity model of Equation (21) results in a notable improvement in 24 h OP accuracy. The predicted RMS position errors decrease by at least 250 m (12%) for Case E and up to 900 m (50%) for Case B. And the Maximum position errors reduce by at least 500 m (16%) for Case C and up to 1950 m (37%) for Case A. In addition, when using Equation (21), both the RMS and maximum position errors for Cases B, C, and D are smaller and almost at the same level. This suggests that refinements in the dynamics model enhance the robustness of OD to the initial state uncertainty within a certain range.

Naturally, as the complexity of the orbital dynamics model increases, the total computational time also rises, mainly due to the additional cost of calculating atmospheric drag acceleration in each iteration. Comparing the computation time for the cases shown in Figure 11 and Figure 17, with identical initial state covariance, the OD process using Equation (21) takes approximately 3 to 5 times longer than that using Equation (4).

## 4. Conclusions

This paper presents an orbit determination (OD) method for non-cooperative Starlink satellites performing continuous low-thrust maneuvers under sparse ground-based observations. The adopted reduced orbital dynamics model includes only the perturbations from the Earth’s non-spherical gravity field and the combined effects of low-thrust propulsion and atmospheric drag, which are represented as a net along-track acceleration. This simplification provides a physically consistent yet computationally efficient representation of the satellite’s motion. The OD algorithm is constructed within a batch estimation framework using an unscented transformation based on an optimized rho-minimum sigma points strategy. Numerical simulations under different levels of measurement noise and initial state uncertainty demonstrate that the proposed method achieves reliable convergence and favorable precision of OD and OP. Statistical analysis across a range of satellites shows that, with a measurement noise level of (100 m, 100 arcsec,100 arcsec), the algorithm converges within 10 iterations and produces 24 h OP results with three-dimensional position RMS errors below 3 km. To further evaluate the proposed OD method, a higher-fidelity orbital dynamics model was used for comparison, where the effects of atmospheric drag are treated as an independent perturbation. The results indicate that adopting the high-fidelity model results in a notable improvement in 24 h OP accuracy and enhances the robustness of OD to the initial state uncertainty within a certain range.

However, several limitations remain. The state covariance initialization is still challenging. Our findings reveal that increased initial uncertainties in position and velocity can help improve the stability of orbit determination. However, this benefit is dependent on maintaining proper scaling with acceleration uncertainty—mismatches here degrade orbit prediction accuracy by 13–49% in our test cases. Further work is still needed on proper state covariance initialization. Moreover, the current dynamics model is limited by the assumption of constant along-track acceleration, which makes it unsuitable for situations where the change rate of orbital altitude varies significantly, such as during large variations in thrust or atmospheric drag. Future efforts will focus on the development of orbital anomaly detection and thrust mode transition schemes to extend the applicability of the method to more general mission profiles.

## Figures and Tables

**Table 1:** The locations of station sites.

Station Number	Longitude (deg)	Latitude (deg)	Altitude (m)
1	75.99 E	39.47 N	0
2	109.5 E	18.4 N	0
3	125.31 E	44.88 N	0

**Table 2:** Simulation results observed from Station 1 for satellite 58831 since 7 February 2024 19:25:42 UTC over a 24 h period.

Arc ID	Starting Time	Ending Time
1	7 February 2024 20:31:42	7 February 2024 20:35:42
2	7 February 2024 22:05:42	7 February 2024 22:12:42
3	7 February 2024 23:42:42	7 February 2024 23:48:12
4	8 February 2024 01:21:42	8 February 2024 01:23:42
5	8 February 2024 02:57:42	8 February 2024 03:02:12
6	8 February 2024 04:32:42	8 February 2024 04:39:42
7	8 February 2024 06:09:42	8 February 2024 07:14:12

**Table 3:** 24 h position errors of OP using different initial covariances of *a*_T_.

Case	RMS Position Error (m)	Maximum Position Error (m)
1-E	1274	2768
1-E′	900	2057
1-F	1395	3085
1-F′	1217	2408
3-E	2080	5143
3-E′	1060	2690
3-F	2308	5725
3-F′	1508	3424

**Table 4:** RMS position errors using different orbital dynamics models.

Dynamics Model	RMS Position Error of 24 h OP (m)					
	A	B	C	D	E	F
Equation (4)	1776	1837	1354	1148	2080	2308
Equation (21)	1380	942	950	820	1835	1911

**Table 5:** Maximum position errors using different orbital dynamics models.

Dynamics Model	Maximum Position Error of 24 h OP (m)					
	A	B	C	D	E	F
Equation (4)	5333	3615	3052	3092	5143	5725
Equation (21)	3383	2645	2541	2074	3946	4160

## Data Availability

Data will be made available upon request.
